# Identification of Small-Molecule Inhibitors for Osteosarcoma Targeted Therapy: Synchronizing *In Silico*, *In Vitro*, and *In Vivo* Analyses

**DOI:** 10.3389/fbioe.2022.921107

**Published:** 2022-06-23

**Authors:** Juan Liu, Qi Yao, Yu Peng, Zhihong Dong, Lu Tang, Xiaoyu Su, Lishuang Liu, Cheng Chen, Murugan Ramalingam, Lijia Cheng

**Affiliations:** ^1^ School of Basic Medical Sciences, Affiliated Hospital, School of Mechanical Engineering, Chengdu University, Chengdu, China; ^2^ Institute of Tissue Regeneration Engineering, Department of Nanobiomedical Science, BK21 NBM Global Research Center for Regenerative Medicine, Dankook University, Cheonan, South Korea

**Keywords:** osteosarcoma, biocomposites, small molecules, virtual screening, targeted therapy

## Abstract

**Objective:** The study aimed to explore a new approach for the treatment of osteosarcoma through combining biomaterials with next-generation small molecule–based targeted therapy.

**Methods:** The model of osteosarcoma was established by 4-hydroxyaminoquinoline 1-oxide (4-HAQO) in mice while the collagen-thermosensitive hydrogel–calcium phosphate (CTC) biocomposites were prepared, and the small molecule inhibitors were virtually screened and synthesized. Then, for the osteosarcoma cell line, MG-63 cells were used to validate our bioinformatic findings *in vitro*, and the mouse osteosarcoma models were treated by combing CTC composites and small-molecule inhibitors after debridement.

**Results:** Five compounds, namely, ZINC150338698, ZINC14768621, ZINC4217203, ZINC169291448, and ZINC85537017, were found in the ZINK database. Finally, ZINC150338698 was selected for chemical synthesis and experimental verification. The results of the MTT assay and Hoechst staining showed that the small-molecule inhibitor ZINC150338698 could significantly induce MG-63 cell death. Furthermore, CTC composites and ZINC150338698 could repair the bone defects well after the debridement of osteosarcoma. In addition, the biomaterials and small-molecule inhibitors have good biocompatibility and biosafety.

**Conclusion:** Our findings not only offer systems biology approach-based drug target identification but also provide new clues for developing novel treatment methods for future osteosarcoma research.

## Introduction

Osteosarcoma is the most common primary bone cancer in children and adolescents and the third most common in adults (Czarnecka et al., 2020). With the development of surgery and chemotherapy, the survival rate of osteosarcoma patients without distant metastasis has been greatly improved. However, although the treatment of osteosarcoma has improved in the past three decades, the overall survival rate of patients has reached a plateau, and about 30–40% of the patients experienced progressive metastasis and died within 5 years after diagnosis (Fernandez-Pineda et al., 2011; Liao et al., 2019). Therefore, it is necessary to find new biomarkers and molecular therapeutic targets for osteosarcoma.

Targeted therapy, also referred to as precision medicine, is a relatively new type of cancer treatment, particularly osteosarcoma, and currently, there is tremendous progress toward the development of targeted drugs. The targeted therapy for osteosarcoma using small-molecule drugs would conceptually be more specific than the conventional non-targeted therapy, such as chemotherapy, radiation therapy, and surgical treatment. The reason is that the small-molecule drugs are small enough to enter the targeted cancer cells, but not the normal cells, and can block the pathway that is responsible for the cancer cells to multiply and spread.

In the past decade, molecular therapeutic targets for bone cancer were identified as individual gene products, and various systems biology approaches are being employed that involve unbiased genome sequencing. DAVID is a Database for Annotation, Visualization, and Integrated Discovery (Dennis et al., 2003). GO (Gene Ontology) and KEGG (Kyoto Encyclopedia of Genes and Genomes) pathway analyses can be performed in DAVID. The STRING database is the network and enrichment facilities in STRING that enable the comprehensive characterization of user gene lists and functional genomics datasets and allow the creation of protein–protein interaction (PPI) networks (Szklarczyk et al., 2021). In this study, we selected two microarray datasets (GSE12865 and GSE36001) from the GEO (Gene Expression Omnibus) database for analysis and screened out differentially expressed genes (DEGs) between osteosarcoma patients and normal people. Through comprehensive analysis in DAVID and STRING databases, the key genes of osteosarcoma were obtained. Then, molecular docking was used to screen out the small-molecule inhibitors that may inhibit the growth of osteosarcoma.

In the conventional treatment of osteosarcoma, the tumor part is surgically removed. The large segment of bone defects left behind by surgical debridement would not heal on their own and require bone grafts to be filled (Dang et al., 2020; Feder et al., 2020). There are a variety of biomaterials that have been used for bone repair and regenerative applications. Calcium phosphate-based composite is one of the effective biomaterials with wide application prospects (Schweikle et al., 2019). Our previous experiments have proved that collagen-thermosensitive hydrogel–calcium phosphate (CTC) bio-composites have excellent osteoinductivity, osteoconductivity, and biological activity, which could be used for load-bearing bone repair. Furthermore, we also have previously demonstrated that the osteogenic ability of CTC is stronger than that of traditional calcium phosphate biomaterials, such as hydroxyapatite/tricalcium phosphate (HA/TCP) (Cheng et al., 2021). Keeping these points in view, in this study, we explored a new approach of targeted therapy by combining surgery, biomaterials with small-molecule inhibitors by database screening, which could treat the mouse osteosarcoma constructed with 4-hydroxyaminoquinoline 1-oxide (4-HAQO). This study may open a new avenue and direct the path for developing novel treatment methods for future osteosarcoma research.

## Materials and Methods

### Mouse Osteosarcoma Model Construction

Twenty BALB/c mice were purchased from Dossy Biological Technology Company (Chengdu, China). All animals were maintained in a temperature and light-controlled environment ventilated with filtered air. All animals were anesthetized with an intraperitoneal injection of pentobarbital sodium. The hair on both legs was removed using an electric shaver, and the skin underneath was disinfected with 75% ethanol, and then, the skin and muscle were cut open, and the tibia was exposed; then, an incision was made to the tibia to expose the marrow cavity; next, 1 mg 4-HAQO was placed into the incision of the bone marrow with a small scraper in the right leg of the mouse, and the left leg had the tibia cut off without any drugs in the same mouse. Twelve weeks later, the osteosarcoma models were identified by histological staining and used for subsequent treatment. The study is reported in accordance with Animal Research: Reporting of *In Vivo* Experiments (ARRIVE) guidelines version 2.0 (Percie du Sert et al., 2020). The Animal Care and Use Committee of Chengdu University approved the study. The operative procedures and animal care were performed in compliance with ARRIVE guidelines on the care and use of laboratory animals, under the supervision of a licensed veterinarian.

### Targets Screening of Key Genes of Osteosarcoma

**FIGURE 1:**
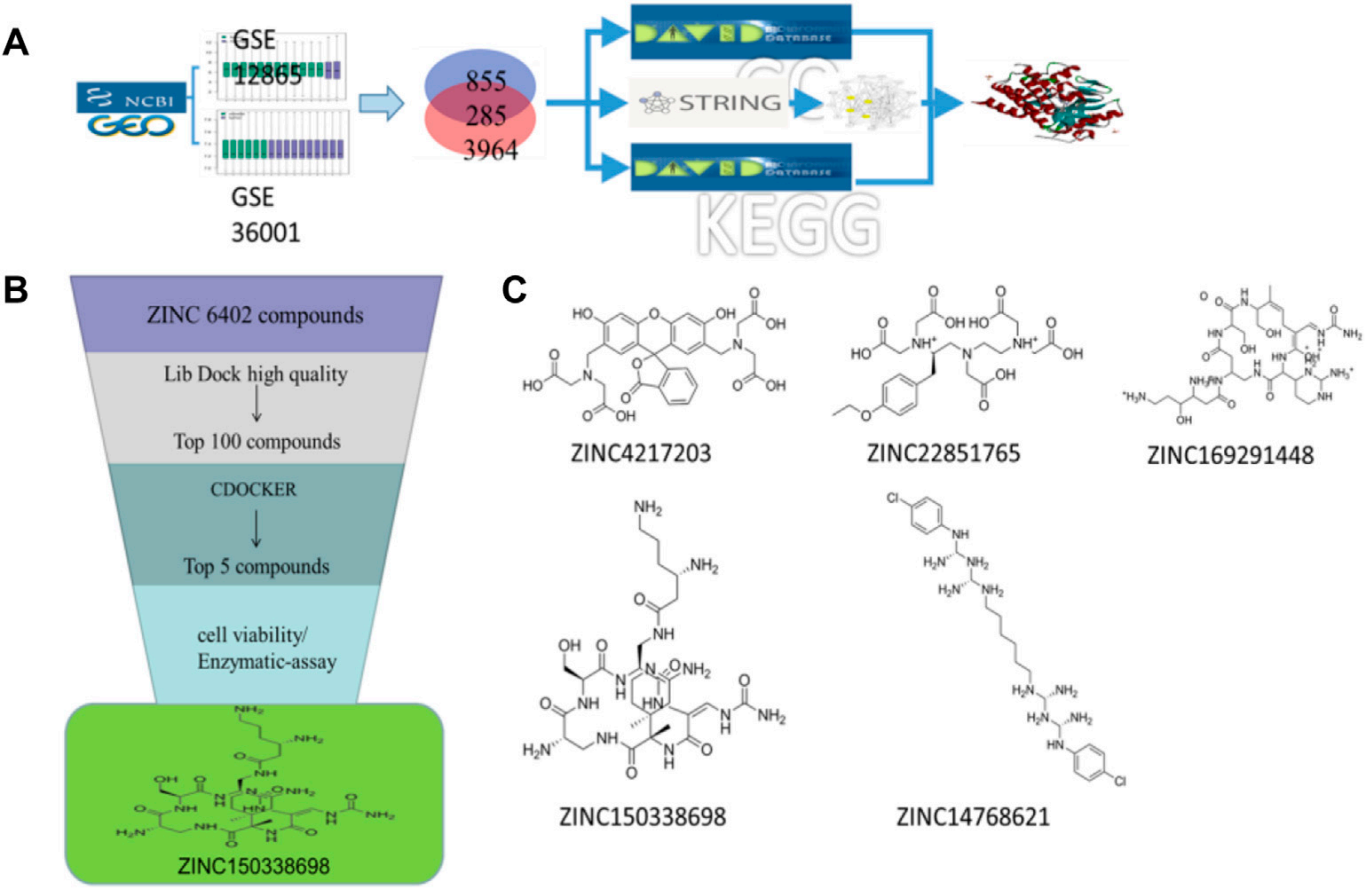
**(A)** Schematic diagram of screening key genes; **(B)** virtual docking process; **(C)** top five compounds in virtual screening results.

Two sets of microarray data from the GEO database set were selected, including GSE12865 and GSE36001. The selection criteria were as follows: 1) osteosarcoma; 2) *Homo sapiens*; 3) tissue or cells containing both normal and tumor types; and 4) DNA copy. GSE36001 was based on the GPL6102 platform (Illumina Human-6 v2.0 Expression Beadchip) and contained 19 osteosarcoma samples and six normal samples (Sadikovic et al., 2009). GSE12865 was based on the GPL6244 platform {(HuGene-1_0-st) Affymetrix Human Gene 1.0 ST Array [transcript (gene) version]} and contained 12 osteosarcoma samples and two osteoblast samples (Kresse et al., 2012). GEO2R (https://www.ncbi.nlm.nih.gov/geo/geo2r/) analysis was used to obtain differentially expressed genes (DEGs). The |LogFC| > 1 and *p* < 0.05 were set as DEG cutoff criteria. Gene ontology (GO) and KEGG pathway enrichment analyses were performed using the DAVID (version 6.8) online tool (https://david.ncifcrf.gov/) (Dennis et al., 2003). GO can be applied to the functions of genes and proteins in all organisms, and there are mainly three types: biological processes, molecular functions, and cellular components. Next, construction and analysis of protein-prtein interaction (PPI) networks were performed by STRING. As shown in Figure 1A, the PPI network was visualized by Cytoscape software, and the core network was identified by the MCODE module. Through DAVID and STRING databases, the key genes of osteosarcoma were screened out.

### Molecular Docking and Screening of Candidate Compounds

The 3D X-ray crystal structure of CSF1R (PDB ID: 6T2W) was downloaded from the RCSB protein database (http://www.rcsb.org/). A total of 6403 candidate compounds were collected from the subset world of the ZINK database (https://zinc.docking.org/substances/subsets/world/). Protein 6T2W (receptor) and these compounds (ligands) were used in molecular docking studies by Discovery Studio (DS) to evaluate binding affinities between them. In DS, LibDOCK and CDOCKER programs were adopted for molecular docking for semi-flexible docking. The binding site of the known ligand (Ligand1) in the 6T2W structure is selected and defined as an active pocket, the ligand molecules are prepared, the energy of compounds is minimized, and the LibDOCK program is used for preliminary docking, and the LibDOCK score is an index to evaluate docking results (Figure 1B). The top 100 small molecules are selected with the highest scores and different structures; the five compounds with the highest LibDOCK score are selected for the same molecule with different conformations (Figure 1C). Next, a second docking was performed using the CDOCKER program provided in DS, and CDOCKER INTERACTION ENERGY evaluated the effectiveness of the docking of different chemicals. CDOCKER ENERGY is best preserved for different conformations of the same compound. In this program, the molecular docking method based on CHARMM was used to dock the ligand with the protein-binding site. When high-temperature molecular dynamics (MD) is used, CDOCKER allows a single protein target to be finely docked to a large number of ligands, resulting in a random ligand conformation. Last, the compound ZINC150338698 with the best CDOCKER INTERACTION ENERGY was selected for chemical synthesis.

### Cell Culture

MG-63 cells were purchased from American Type Culture Collection (ATCC, Manassas, VA, United States). The cells were cultured in Dulbecco’s modified Eagle medium (DMEM) with 10% fetal bovine serum (FBS) and 1% penicillin/streptomycin and maintained in a 5% CO_2_/37°C incubator, respectively. Cells were used with 0.25% trypsin, DMEM, FBS, and antibiotics were purchased from Gibco (Carlsbad, CA, United States).

### (4,5-Dimethyl-2-Thiazolyl)-2,5-Diphenyltetrazolium Bromide (MTT) Assay

For the assay, 5×10^3^ MG-63 cells were seeded in a 96-well plate, respectively. The small molecule inhibitor ZINC150338698 is dissolved in anhydrous ethanol and then diluted with normal saline. Eighteen hours post-seeding, cells were treated with different concentrations of ZINC150338698 as follows: 0.1 μmol/L, 1 μmol/L, and 10 μmol/L ZINC150338698. Cisplatin was used as a positive control, and untreated cells were set as blank control. Each group was set with three wells. Then, 48 h post drug treatments, the viable cells were stained by adding 20 μl of 5 mg/ml MTT solution per 100 μl of growth medium. After incubating for 4 h at 37°C, the media were removed, and 150 μl DMSO was added to dissolve the formazan. The absorbance of each well was measured by using a microplate reader, and viable cells are presented as a percentage of the control.

### Hoechst Staining

For staining, 1×10^5^ MG-63 cells were seeded in a 6-well plate. The cells were treated with different concentrations of small-molecule ZINC150338698 as follows: 0.1 μmol/L, 1 μmol/L, and 10 μmol/L ZINC150338698 after 18 h. Cisplatin was used as positive control, and untreated cells were set as blank control. Each group was set with three wells. Then, 48 h later, cells were stained with a Hoechst kit from Beyotime (Haimen, Jiangsu, China). Cell counting was carried out by ImageJ software from the National Institutes of Health, which is available at http://rsbweb.nih.gov. The corresponding cell death rates were calculated according to Hoechst staining.

### Western Blot

The cells were used to extract proteins and then were separated using a 10% polyacrylamide gel. After transferring the protein on a nitrocellulose membrane, the membrane was blocked with a 5% defatted milk solution and probed with the mouse monoclonal antibody against CSF1R (1:2000, Abcam) and GAPDH (1:5000, Chemicon) and then probed with a secondary antibody using ALP-conjugated anti-mouse IgG (1:5000, Chemicon). Last, blots were developed using an ECL Plus kit (GE, United States).

### Preparation of Collagen-Thermosensitive Hydrogel–Calcium Phosphate Composites

**FIGURE 2:**
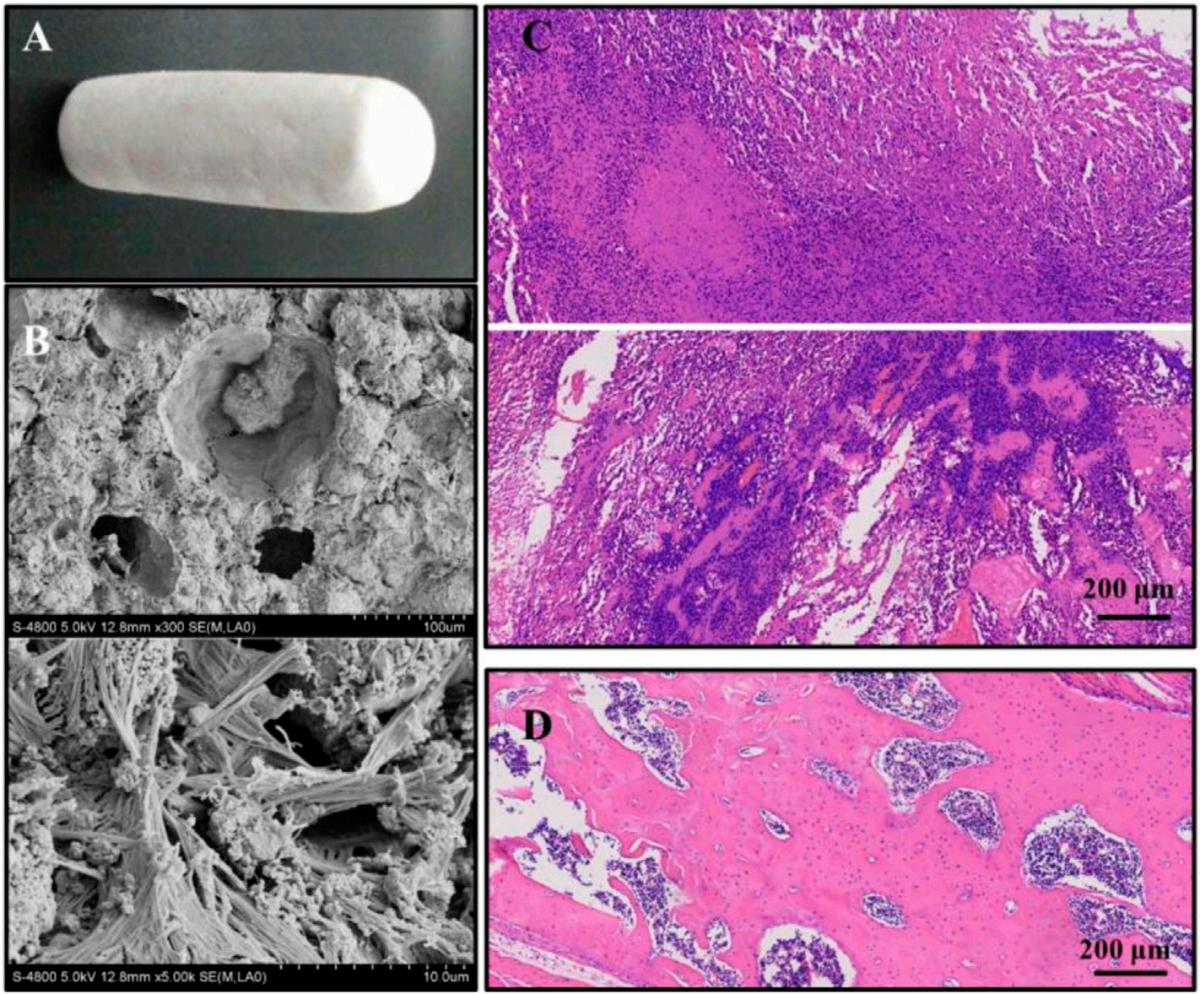
**(A)** Macroscopic structures of collagen-thermosensitive hydrogel-calcium phosphate (CTC) composites; **(B)** microscopic structures by magnification 300 and 5000 times of CTC composites; **(C)** osteosarcoma models were successfully constructed by 4-HAQO, bar: 200 μm; **(D)** control group without tumors, bar: 200 μm.

The bio-composites used in this study were made up of thermosensitive hydrogel using type I collagen and tricalcium phosphate powder. The ratio of type I collagen (Sigma-Aldrich, United States) to hydrogel solution was 15:25 and stirred for 10 min to obtain the mixture of the collagen-thermosensitive hydrogel. Then, the tricalcium phosphate powder [Ca_3_(PO_4_)_2_, Sigma-Aldrich, United States] was weighed and mixed with the collagen-thermosensitive hydrogel solution in a ratio of 40:60 for 10 min. The collagen-thermosensitive hydrogel–calcium phosphate (CTC) mixture was obtained (Figure 2A). The surface structures of the composites were measured by scanning electron microscopy (SEM) (Figure 2B). The composites were fabricated into a shape corresponding to the debridement of osteosarcoma and steam-sterilized at 121°C for 15 min before implantation.

### Surgery and Targeted Therapy for Osteosarcoma

Ten mouse osteosarcoma models were selected and randomly divided into two groups (*n* = 5): the experimental group and the control group. Then all animals were anesthetized with an intraperitoneal injection of pentobarbital sodium. The hair on the right leg was removed using an electric shaver, and the skin underneath was disinfected with 75% ethanol and cut open. All tumor tissues were removed, including osteomas and sarcomas, and the tibia was amputated. In the experimental group, after debridement is completed, the tibial-shaped CTC composite was grafted to the bone defect and simply fixed. The skin was closed with nylon sutures, and penicillin was injected intramuscularly to prevent infection finally. Next, 50 mg/kg ZINC150338698 was injected through the tail vein from the day of the surgery, and then, the same dose was injected every 3 days, a total of 20 administrations. In the control group, there was no material filling and small-molecule inhibitor administration after debridement, the skin was closed, and penicillin was injected intramuscularly as the experimental group. After 60 days, the mice were sacrificed. The complete tibia was harvested and fixed with a 4% formaldehyde solution to be used for follow-up research.

### Detection of the Therapeutic Effect by Imaging

After fixation in the formaldehyde solution, the samples were stored in alcohol and sent to scan μ-CT by a high-resolution Skyscan 1174 (Bruker, Belgium) at 10-μm voxel resolution and 55 kV. For three-dimensional analysis by μ-CT, 400 images of each sample were three-dimensionally reconstructed with the best threshold, and the overall and cross-section images were produced with their own software. In the meantime, the overall osteogenesis and bone trabecula were automatically analyzed through the value of total volume (TV), bone volume (BV), BV/TV, structure model index (SMI), trabecular thickness (Tb.Th), trabecular number (Tb.N), trabecular separation (Tb.Sp), and bone mineral density (BMD).

### Detection of the Therapeutic Effect by Histology

After scanning μ-CT, all samples were decalcified in 10% ethylene diamine tetraacetic acid (EDTA, pH 7.0) for about 20 days at room temperature, dehydrated, and embedded in paraffin. The embedded samples were cut into 5-μm thick histological sections. Then, they were stained with hematoxylin-eosin (HE), Masson-trichrome staining, and tartrate-resistant acid phosphatase staining, according to the manufacturer’s instructions. Finally, all tissue slices were imaged under the microscope and analyzed by image analysis software.

### Statistical Analysis

Data were expressed as means ± standard deviation and analyzed by paired ANOVA (SPSS 13.0, SPSS, United States). *p* < 0.05 was considered statistically significant.

## Results

### Construction of the Osteosarcoma Model

The osteosarcoma models were successfully constructed in 18 of the 20 mice with a success rate of 90% by 4-HAQO. The average tumor volume was 1581 ± 367 mm^3^, and the average tumor weight was 3.89 ± 0.76 g. In the osteosarcoma models, HE images showed obvious nuclear atypia of tumor cells, which were fusiform or polygonal; the multinucleated giant cells were also observed, and some blood vessels were surrounded by tumor cells (Figure 2C), while the fracture had healed and no tumor cells were observed in the control group (Figure 2D).

### Target Gene of Osteosarcoma

**FIGURE 3:**
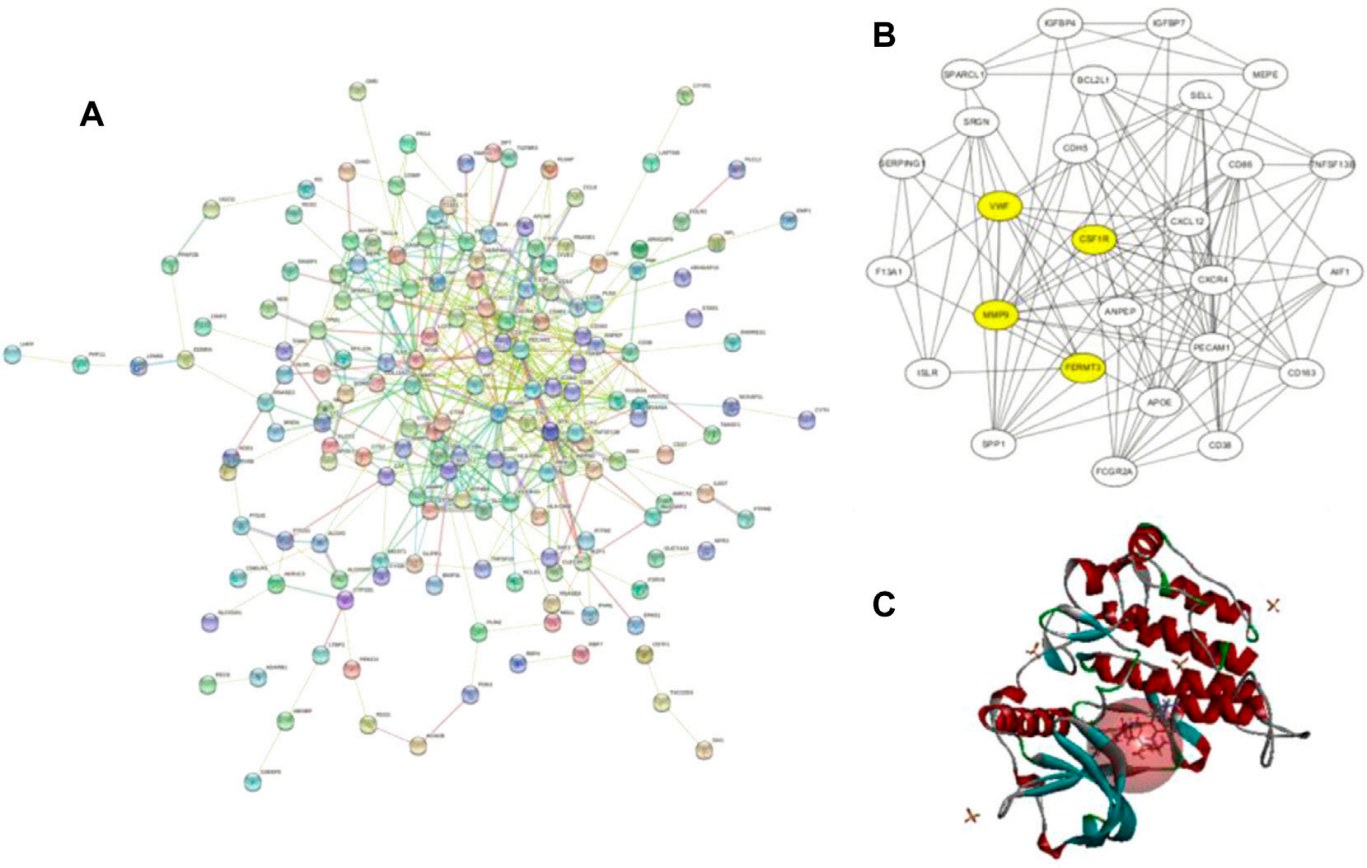
**(A)** Protein–protein interaction network of upregulated genes from the STRING database; **(B)** this interaction network is the core network from **(A)**, which was calculated in Cytoscape. The four yellow core HUB proteins were obtained by comprehensive analysis with KEGG and GO; **(C)** binding diagram of ZINC150338698 to protein 6T2W, and the red ball is the binding site.

A total of 855 and 3964 DEGs were identified from GSE36001 and GSE12865 datasets. In total, 215 genes were screened out in two datasets and were selected for further analysis. The upregulated genes were analyzed by the David 6.8 online tool, and the results of Gene Ontology and KEGG pathway enrichment analyses were obtained, respectively. Meanwhile, we analyzed these upregulated genes through a STRING database to obtain a PPI network (Figure 3A); the core PPI network obtained by Cytoscape software contained 27 genes. Through the comprehensive integration of GO, KEGG pathway analysis, and Cytoscape software analysis results, four osteosarcoma key genes, *MMP9*, *FERMT3*, *CSF1R*, and *VWF*, were finally identified (Figure 3B). There are some studies on the relationship between MMP9, VWF, and osteosarcoma. FERMT3 has barely been studied. In addition, we found some studies on CSF1R in osteosarcoma in the past five years. In the study of Fujiwara et al. (2021), the FDA-approved CSF1R inhibitor PLX3397 can suppress the growth of osteosarcoma. Smeester et al. (2020) found that CSF1R is utilized by OSA cells to promote tumorigenesis. Wen et al. (2017) found that CSF-1R inhibition in osteosarcoma cells by RNA interference suppresses cell proliferation and tumor growth in mice. Finally, we chose to make some contributions to the research on CSF1R in osteosarcoma.

### Screening of CSF1R Inhibitors

The molecular docking of 6,403 compounds collected in the ZINK database with protein 6T2W was studied. Initial docking with the LibDOCK yielded 1,314,664 compounds, with the 100 compounds with the highest retention scores (all with -LibDOCK score >140). The second docking of these compounds by CDOCKER yielded 4,947 compounds. The top five compounds in docking results were ZINC150338698, ZINC14768621, ZINC4217203, ZINC169291448, and ZINC85537017, as shown in Table 1, which might have effects on osteosarcoma. Finally, ZINC150338698, the compound with the best CDOCKER INTERACTION ENERGY, was selected for chemical synthesis and experimental verification. Figure 3C showed the binding diagram of ZINC150338698 to protein 6T2W.

**TABLE 1:** Top five compounds from virtual screening results.

	Name	2D STRACTION	-LibDOCK score	-CDOCKER INTERACTION ENERGY
1	ZINC150338698	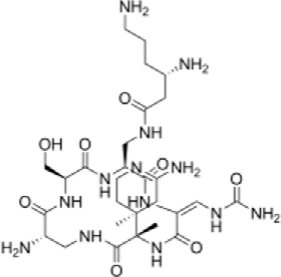	148.955	88.2205
2	ZINC14768621	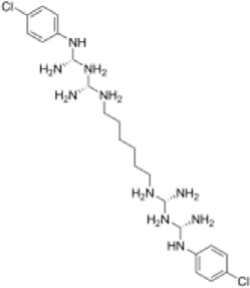	153.232	82.9094
3	ZINC4217203	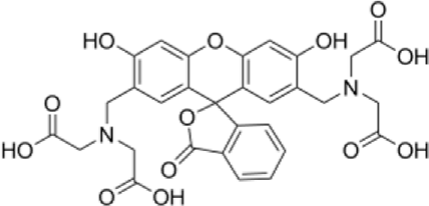	169.416	82.0324
4	ZINC169291448	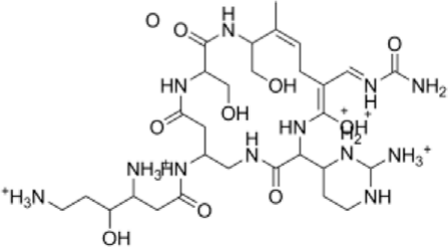	146.643	81.2782
5	ZINC22851765	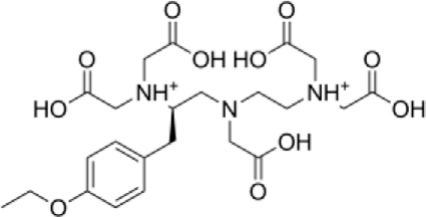	163.706	80.7144

### Effects of the Compound ZINC150338698 on Osteosarcoma Cell Viability

**FIGURE 4:**
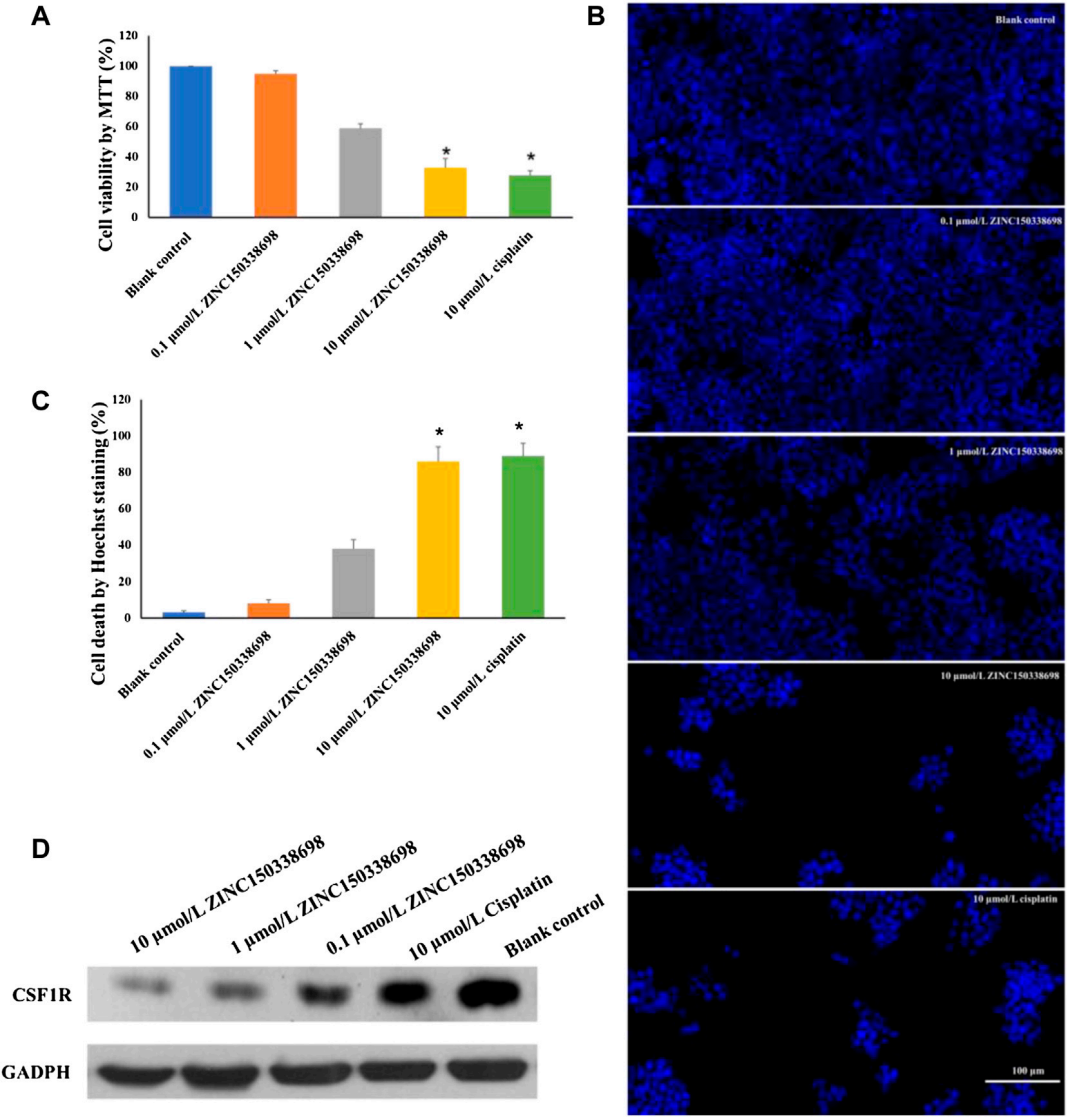
**(A)** Viability of MG-63 cells was detected by MTT assay. The cell growth was significantly inhibited by 10 μmol/L ZINC150338698 at 48 h. **(B)** Percentage of cell death according to Hoechst staining at 48 h. Compared with the blank control group, *p < 0.05. **(C)** Expression of CSF1R in MG-63 cells after treatment at 48 h; GADPH was set as a positive control. **(D)** Corresponding gray-scale value of CSF1R based on GAPDH, compared with the blank control group, *p < 0.05.

To determine whether the treatment of ZINC150338698 affected osteosarcoma cell survival abilities, the authors examined the cell viability by MTT assay after drug treatment for 48 h. The results demonstrated that the cell survival abilities were significantly decreased with 10 μmol/L ZINC150338698 (*p* < 0.05), whose effects were similar to those of the positive control group (Figure 4A). To further investigate the effects of ZINC150338698 on the cell survival rate, the authors investigated the percentages of cell death of MG-63 cells, upon ZINC150338698 treatment by Hoechst staining. Compared to the untreated group, the percentage of dead cells was significantly increased in the group treated with 10 μmol/L ZINC150338698 and cisplatin (Figure 4B). The expression of CSF1R was inhibited by ZINC150338698, especially by 10 μmol/L ZINC150338698; however, cisplatin did not significantly inhibit the expression of CSF1R (Figure 4C). The gray-scale value of each band was converted to the relative expression of CSF1R (Figure 4D, *p < 0.05). These results indicated that ZINC150338698 could effectively inhibit the osteosarcoma cell survival through the inhibition of CSF1R.

### New Bone Formation After Treatment

**FIGURE 5:**
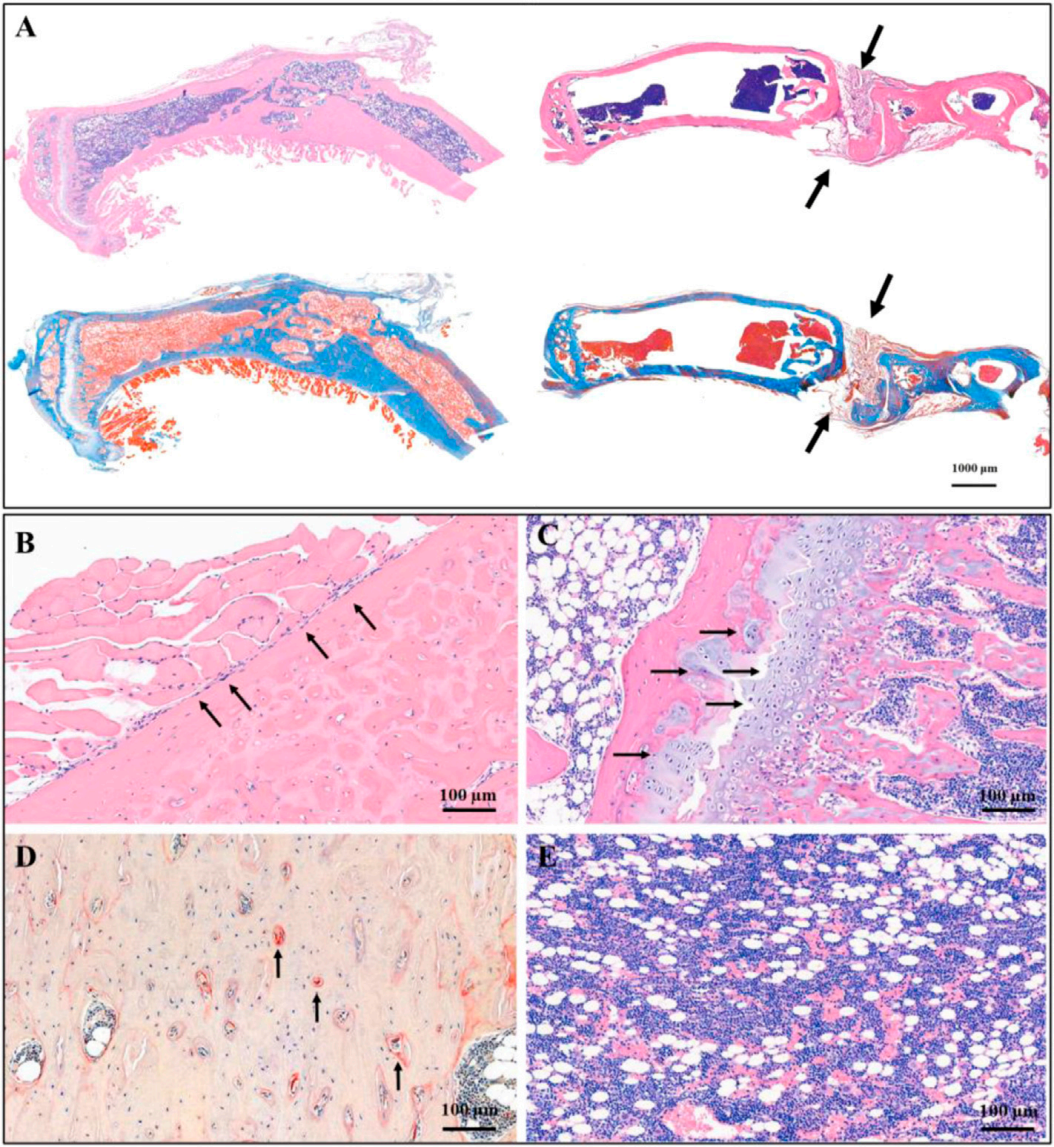
Histological staining showed the therapeutic effect of CTC bio-composite engraftment and ZINC150338698 administration on osteosarcoma. **(A)** HE and Masson trichromatic staining showing the entire tibia in the experimental group (left) and the control group (right); the bone defect healed well in the experimental group, while the bone defect showed nonunion in the control group. Arrow: the unhealed bone defect; bar: 1000 μm. **(B)** In the area of new bone formation, the osteoblasts lined up around the new bone tissues by HE staining, arrow: osteoblasts; bar: 100 μm. **(C)** Growth of chondrocytes was detected by HE staining, indicating that the new bone is formed by endochondral ossification. Arrow: chondrocytes; bar: 100 μm. **(D)** Osteoclasts were determined by tartrate-resistant acid phosphatase staining, indicating the dynamic balance between osteoblasts and osteoclasts. Arrow: osteoclasts; bar: 100 μm. **(E)** Mature bone marrow tissue appeared in the marrow cavity. Bar: 100 μm.

The osteosarcoma models were successfully constructed by 4-HAQO, to treat osteosarcoma, a combination of surgery, biomaterial filling, and targeted therapy was used. Two months later, the results showed that the removed tibia was replaced by new bone tissue, which was induced by the CTC composites, and the debridement defects healed well in the experimental group, while the results showed the bone nonunion at the debridement site, and the new bone tissue grew irregularly in the control group (Figure 5A). We also found a balanced and dynamic growth of osteoblasts (Figure 5B), chondrocytes (Figure 5C), osteoclasts (tartrate-resistant acid phosphatase staining, Figure 5D), and marrow cells (Figure 5E) in the experimental group.

### Evaluation of the Postoperative Recovery

**FIGURE 6:**
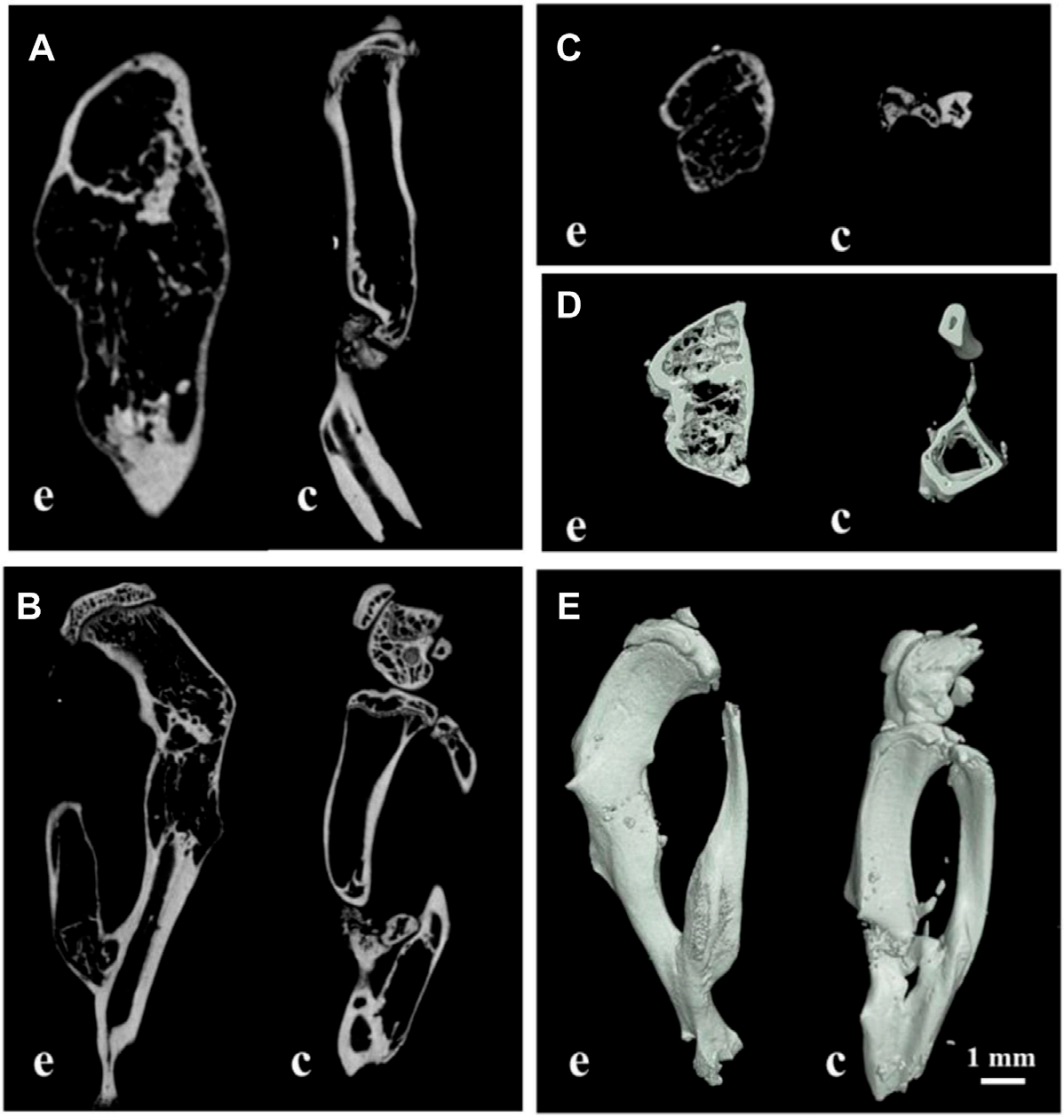
Multi-view of sagittal **(A)**, transverse **(B)**, coronal **(C)**, and sectional planes **(D),** and the stereogram **(E)** of mouse tibia by micro-CT; **(E)** the experimental group; **(C)** the control group; bar: 1 mm.

To reconstruct the real scene of bone healing *in vivo*, the micro-CT was used to reveal the specific and visualized repair results 60 days after surgery. The multi-view of sagittal, coronal, and transverse planes, and the stereogram was captured to show the details. From these pictures, it could be seen that the osteosarcoma was well treated, and no unhealed bone defect remained in the experimental group, while there is a bone nonunion in the control group (Figure 6). The results of micro-CT are consistent with those of histological staining. According to the results of micro-CT, the data of osteogenesis and trabecula were analyzed with built-in software of micro-CT in the experimental group and control group, and the unoperated control group was set as a reference (Table 2), for further analysis of therapeutic effects.

**TABLE 2:** Parameters of osteogenesis in bone healing.

	TV (mm^3^)	BV (mm^3^)	BV/TV (%)	SMI	Tb.Th (mm)	Tb.N (1/mm)	Tb.Sp (mm)	BMD (g/Cm^3^)
Experimental group	18.3522 ± 0.3515	10.2593 ± 0.5796	0.5590 ± 0.0357	1.7965 ± 0.0593	0.1676 ± 0.0136*	2.1551 ± 0.1157*	0.6549 ± 0.0527	1.1546 ± 0.1654*
Control group	21.1975 ± 0.3675	8.5345 ± 0.2707	0.4026 ± 0.0091	2.3477 ± 0.0883	0.1175 ± 0.0070	1.1223 ± 0.0302	0.7019 ± 0.0305	0.6967 ± 0.0173
Unoperated control group	21.4892 ± 0.9957	14.7635 ± 0.6580	0.6870 ± 0.0430	1.6034 ± 0.0243	0.1782 ± 0.0094	2.8746 ± 0.1344	0.6012 ± 0.0442	1.6531 ± 0.1165

The comparison between the experimental group and control group, *p < 0.05. TV: tissue volume; BV: bone volume; BV/TV: bone volume fraction; SMI: structural mode index; Tb.Th: trabecular thickness; Tb.N: trabecular number; Tb.Sp: trabecular separation; BMD: bone mineral density.

### Biological Safety of Materials and Small-Molecule Inhibitors

**FIGURE 7:**
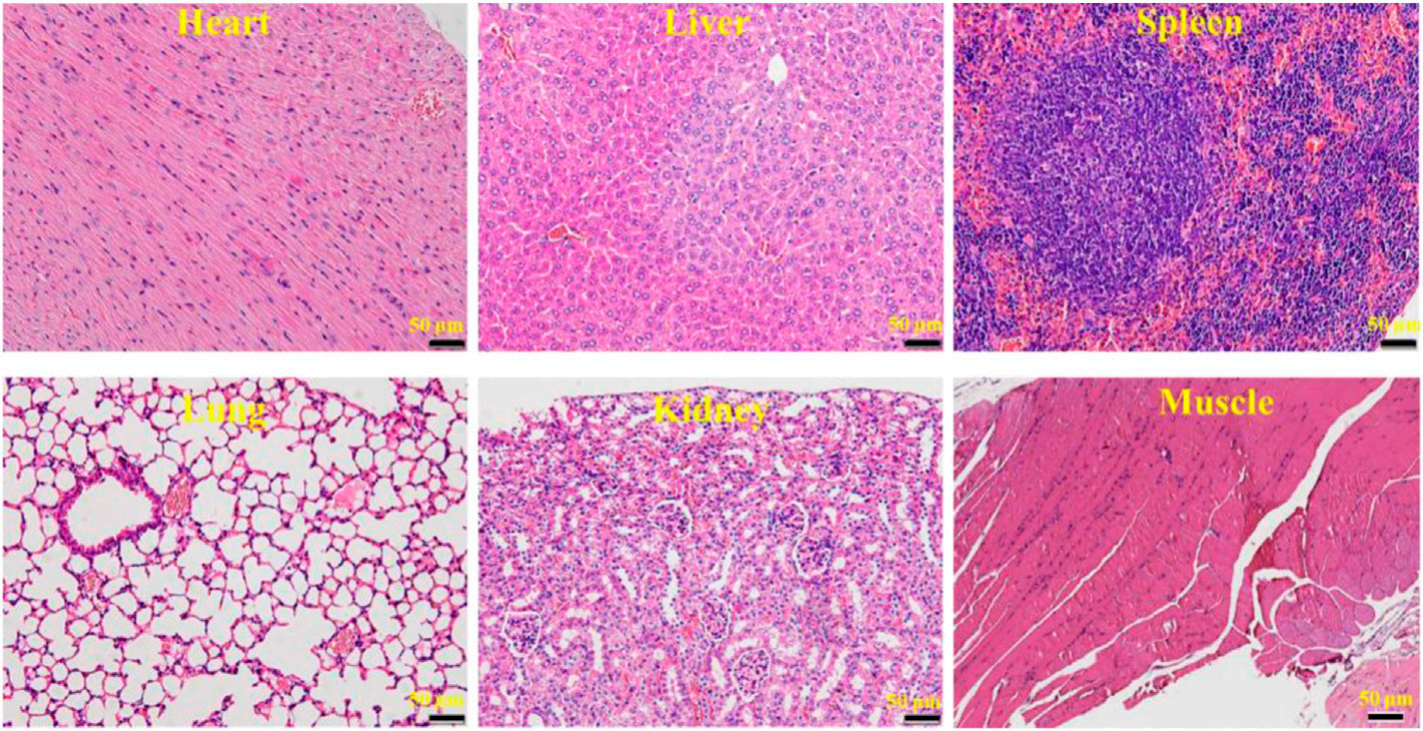
HE micrograph showing no inflammation, foreign body, or tumor tissue in the heart, liver, spleen, lung, kidney, and the muscle tissues adjacent to cancer tissues. Bar: 50 μm.

After treatment of osteosarcoma with a combination of CTC composites and compound ZINC150338698, to detect the side effects of the biomaterials and small-molecule inhibitors, the authors examined the microscopic structure of the heart, liver, spleen, lung, kidney, and the muscle tissues adjacent to cancer tissues in the experimental group and the control group. The results showed that no inflammation, foreign body, or tumor tissue was observed in the two groups (Figure 7), which suggested our biomaterials and small-molecule inhibitors have good biocompatibility and biosafety.

## Discussion

Osteosarcoma is the most common primary malignant bone tumor; the highest incidence is in children and adolescents (median age of 18) (Corre et al., 2020). These tumors occur mainly in the long bones and less frequently in the skull, jaw, and pelvis. Nowadays, the best treatment options for osteosarcoma include chemotherapy, surgery, radiotherapy, and immunotherapy (Lussier et al., 2015; Yu et al., 2019; Hız et al., 2021); however, the optimal scheme has not yet been defined. Surgical treatment was fundamental, and the complete surgical resection of all sites of tumor tissues remains essential for survival (Kager et al., 2017). Over the past few decades, the prognosis of metastatic and relapsed osteosarcoma has remained stagnant, although several anticancer drugs have been clinically applied (Thanindratarn et al., 2019); the metastatic rate of osteosarcoma is as high as 40% within 5 years after diagnosis, metastasis to the lungs is common, but also to the liver and lymph. To date, new treatment options for osteosarcoma, especially relapsed and metastatic osteosarcoma, are still limited (Chen et al., 2021). Therefore, the development of appropriately targeted therapy drugs may effectively inhibit the metastasis of osteosarcoma.

In the study, by the method of bioinformatics analysis, the possible key genes of osteosarcoma including MMP9, FERMT3, CSF1R, and VWF were screened from two microarray datasets, GSE12865 and GSE36001. These four key genes may be potential targets for the diagnosis and treatment of osteosarcoma. Through consulting relevant literature reports, the four genes were analyzed, and finally, CSF1R was selected for subsequent research. CSF1R is a member of the receptor protein tyrosine kinase (rPTK) family of growth factor receptors (Ries et al., 2014). It has been proved that the infiltration of tumor-associated macrophages (TAMs) was related to the driving force of tumorigenesis and the suppression of antitumor immunity. CSF1R is a cellular receptor for colony-stimulating factor-1 (CSF-1) and interleukin-34 (IL-34), which plays a nuclear role in the manipulation of TAMs (Holmgaard et al., 2016; Xun et al., 2020). In this context, various approaches targeting either the ligands or the receptor are currently in clinical development. His existing inhibitors for CSF1R include PLX3397 and RG7155 (Ries et al., 2014). The FDA-approved CSF1R inhibitor PLX3397 can suppress the growth of osteosarcoma in mice (Fujiwara et al., 2021). We did not find a correlation between RG7155 and osteosarcoma. The protein structure expressed by CSF1R is subjected to molecular docking with compounds downloaded by ZINC in Discovery Studio. ZINC150338698 showed the best docking results when analyzed by LibDOCK and CDOCKER. Therefore, we chose ZINC150338698 small-molecule inhibitors for osteosarcoma *in vitro* and *in vivo*. Our results showed that the small-molecule inhibitor ZINC150338698 could effectively inhibit the proliferation of osteosarcoma cells *in vitro* and promote bone repair after surgery *in vivo*. However, the postoperative evaluation time is too short to directly indicate the effect of targeted therapy, and we will extend the evaluation time in future studies.

Most patients with osteosarcoma required surgical amputation to cure the tumor completely, which needed a bone graft (Zhao et al., 2020; Barr and Howe, 2018; El Beaino et al., 2019). At present, the most advantageous bone graft material was the artificial bone compared to autogenous and allogeneic bone grafts. Calcium phosphate–based composites were the focus of research in the department of orthopedics, stomatology, and plastic surgery in recent years, which could achieve the same effect of autologous bone transplantation with the advantages of economic, wide source, and no immunogenicity (Richter et al., 2019; Birkholz et al., 2016; Waiyawat et al., 2020; Ma et al., 2019). HE and Masson staining showed that the tibia was replaced by new bone tissue induced by the CTC composite. Tricalcium phosphate of CTC composites could degrade to form a hydroxyapatite layer and then serve as a cell scaffold to promote the proliferation of osteoblasts and detect the growth of chondrocytes; however, it needs to be combined with other substances to become osteoinductive, so the addition of type I collagen and temperature-sensitive hydrogel increases the mechanical properties of tricalcium phosphate and enhances its osteogenic properties. Micro-CT and histological staining showed that the material degraded in mice and induced tibial healing. We can speculate that the degradation rate of CTC material in mice is the same as that of bone healing and repair, thus ensuring complete tibial healing. In addition, it can also be inferred that the properties of CTC composites are stable and have no side effects in mice since there was no significant immune response and inflammatory reaction in the heart, liver, spleen, lung, kidney, and the muscle tissues adjacent to cancer tissues. Micro-CT scanning is an effective way to evaluate the effect of bone repair (Kim et al., 2021; Oliviero et al., 2019); the values of TV, BV, BV/TV, SMI, Tb.Th, Tb.N, Tb.Sp, and BMD are important indicators for the evaluation of osteogenesis. Through the micro-CT, we observed the more trabecular and cortical bone formation and restoration of basic structure in the area of bone defects compared to the control group. In addition, BV/TV has been used to measure tumor-induced osteolysis (Ohba et al., 2014), we can also find that the value in Table 2 demonstrates decreased trabecular osteolysis and increased BMD in the experimental group, which showed that the material degraded in mice and induced tibial healing, and the bone repairing effects were consistent with our previous study (Cheng et al., 2021), which indicated that our CTC composites and small-molecule inhibitors have good effects for the treatment of mouse osteosarcoma.

Therefore, the CTC bio-composites were used to fill the defects caused by amputation, and the results showed the new bone tissues induced by the biomaterials repaired the bone defects very well. It is believed that the biomaterials with excellent biological and osteogenic properties, combined with targeted therapy, might effectively reduce the recurrence rate and death rate of osteosarcoma, and the authors will continue to report the related research studies in the future studies.

## Conclusion

In this study, differentially expressed genes (DEGs) from two microarray datasets were identified. Then, the up-regulated gene was analyzed by KEGG, GO, and STRING methods. We chose CSF1R as the receptor for molecular docking, and ZINC150338698 showed the best binding result. In addition, we performed *in vitro* and *in vivo* experiments on the effects of ZINC150338698 in mice. A tibia with osteosarcoma established by 4-HAQO was surgically removed, and the defect was filled with CTC biomaterial and our synthetic inhibitor. Experiments proved that the inhibitor ZINC150338698 could inhibit the growth of osteosarcoma cells, and the bone defects could be repaired by the CTC bio-composite and ZINC150338698. There was no inflammation, foreign body, or tumor tissue observed in mice, suggesting that our biomaterial and small molecule inhibitor had good biocompatibility and biosafety. Our study may be developed as a new candidate drug for the treatment of osteosarcoma in the future.

## Data Availability

The original contributions presented in the study are included in the article/supplementary material; further inquiries can be directed to the corresponding authors.
